# Oral squamous carcinoma cell lysates provoke exacerbated inflammatory response in gingival fibroblasts

**DOI:** 10.1007/s00784-023-05107-x

**Published:** 2023-06-30

**Authors:** Mariane Beatriz Sordi, Layla Panahipour, Reinhard Gruber

**Affiliations:** 1 Department of Oral Biology, University Clinic of Dentistry, Medical University of Vienna, Sensengasse 2a, 1090 Vienna, Austria; 2 Department of Dentistry, Federal University of Santa Catarina, Florianopolis, Brazil; 3 Department of Periodontology, School of Dental Medicine, University of Bern, Bern, Switzerland

**Keywords:** Cell lysates, Necrotic cells, Inflammation, Cytokines, Oral cells, Periodontium

## Abstract

**Objectives:**

To study whether damaged epithelial cells and gingival fibroblast could affect the expression of inflammatory cytokines in healthy cells.

**Materials and methods:**

Cell suspensions were submitted to different treatments to obtain the lysates: no treatment (supernatant control), sonication, and freeze/thawing. All treatments were centrifuged, and the supernatants of the lysates were used for experimentation. Cell viability assays, RT-qPCR of IL1, IL6 and IL8, IL6 immunoassay, and immunofluorescence of NF-kB p65 were applied to verify the inflammatory crosstalk of damaged cells over healthy plated cells. Furthermore, titanium discs and collagen membranes were treated with lysates and checked for IL8 expression by RT-qPCR.

**Results:**

Lysates obtained upon sonication or freeze/thawing of oral squamous carcinoma cell lines provoked a robust increase in the expression of IL1, IL6, and IL8 by gingival fibroblasts, which was confirmed by IL6 immunoassays. Lysates obtained from the gingival fibroblasts failed to increase the expression of inflammatory cytokines in oral squamous carcinoma cells. Additionally, oral squamous carcinoma cell lysates caused the activation of the NF-kB signalling cascade in gingival fibroblasts as indicated by the phosphorylation and nuclear translocation of p65. Finally, oral squamous carcinoma cell lysates adhered to the titanium and collagen membrane surfaces and increased IL8 expression by gingival fibroblasts growing in these materials.

**Conclusions:**

Injured oral epithelial cells can release factors that incite gingival fibroblasts to become pro-inflammatory.

**Clinical relevance:**

Injuries affecting the oral mucosa generate epithelial fragments that may reach the underlying connective tissue and provoke inflammation. These injuries are routinely caused by mastication, sonication for teeth cleaning, teeth preparation, prostheses maladaptation, and implant drilling.

**Supplementary Information:**

The online version contains supplementary material available at 10.1007/s00784-023-05107-x.

## Introduction

The protective periodontium, basically consisted of gingival tissue, provides a vital seal concerning the contaminated oral cavity [1]. The periodontal soft tissues consist fundamentally of (1) the connective tissue, rich in gingival fibroblasts and the respective extracellular matrix, and (2) the oral epithelium surrounding the connective tissue and offering a barrier towards the septic environment and mechanical loads [2, 3]. The integrity of the multilayer epithelium with its cobblestone appearance [4] serving as a biological barrier is mandatory to tighten up the periodontal tissue and prevent saliva components, including the oral microbiota, from reaching the underlying connective tissue rich in gingival fibroblasts and resident macrophages [3, 5]. Nevertheless, the loss of integrity of the oral epithelium is associated with a strong infiltration of the connective tissue with inflammatory cells recruited in response to the cytokines and chemokines released by the connective tissue cells [6, 7]. This principle is extended to other tissues, for instance, the disruption of the epithelial barrier as a potential cause of inflammatory bowel disease [8]. In the oral cavity, mechanical invasive procedures can impair the oral epithelium, especially when causing the disintegration of the epithelial cells per se.

Constant impairment of the physiological epithelial barrier can occur in response to mastication or iatrogenic damage of treatment-related issues, leading to the disruption of the epithelial seal and the concomitant connective tissue inflammation. Ongoing mechanical damage, via induction of IL6 from epithelial cells, tailored effector T cell function, promoting increases in gingival Th17 cell numbers; in other words, mechanical injury triggers immune responsiveness [9]. In this sense, apart from the physiological mechanical damage from mastication, injuries affecting the oral epithelium may be caused by prostheses maladaptation [10], implant drilling [11], teeth preparation [12], or even due to the use of curettes, scalers, sonication, or air polishing for teeth cleaning [13]. Furthermore, some oral pathologies, such as oral carcinoma, may produce epithelial cell fragmentation [14]. Epithelial cell fragments may in turn reach the subjacent gingival fibroblasts of the connective tissue and, hypothetically, provoke an inflammatory response to support the tissue homeostasis [6, 15]. To simulate the situation where connective tissue fibroblasts are exposed to the epithelial cell fragments released from injured cells in vitro, we used lysates of disrupted cells over healthy growing cells.

Lysates of cells can be used to study how damaged tissues activate the immune system and thereby initiate repair and remodelling mechanisms. For instance, cell lysates produced by freeze/thawing odontoblast-like cells caused THP-1 macrophages to produce inflammatory cytokines and angiogenic growth factors [16]. The underlying molecular mechanisms may involve the release of damage-associated molecular patterns (DAMPs) functioning as adjuvants or danger signals for the local cells to play a role in homeostatic mechanisms [6, 7, 17]. This concept was extended towards priming dendritic cells with cancer cell-derived lysates as an immunogenic source for cancer vaccine design [18]. Moreover, melanoma patients were vaccinated with dendritic cells pulsed with cell lysate derived from three melanoma cell lines, thus evoking anti-melanoma immune response [19]. It is complex, however, to understand how DAMPs contribute to maintain periodontal integrity upon indulgence and damage. Nevertheless, there are indirect signs of the release of DAMPs in periodontitis based on the immunostaining of chronic periodontitis [20]. Presumably, not only the virulence factors of bacteria but also oral epithelial cells can become a source of damaging signals. However, whether gingival fibroblast can sense oral epithelial lysates and respond with an inflammatory response remains unknown. Thus, the aim of this in vitro study is to determine if necrotic oral epithelial cells and gingival fibroblast can provoke inflammatory response, which could clinically lead to soft tissue disruption and periodontal disease.

## Material and methods

### Cells

Human gingival fibroblasts (HGF) were prepared from explant cultures of three independent donors after approval of the Ethical Committee of the Medical University of Vienna (EK Nr. 631/2007). Oral squamous cell carcinoma cell line (HSC2), originally obtained from Health Science Research Resources Bank (Sennan, Japan), was kindly provided by Xiaohui Rausch-Fan, Department of Periodontology, Medical University of Vienna, Austria. Oral squamous cell carcinoma from buccal mucosa cell line (TR146) from the European Collection of Authenticated Cell Cultures (ECACC) was kindly provided by Winfried Neuhaus from the Competence Unit Molecular Diagnostics, Center Health and Bioresources, Austrian Institute of Technology (AIT) GmbH. All cells were cultured in a humidified atmosphere at 37 °C in a growth medium consisting of Dulbecco’s Modified Eagle Medium (DMEM, Sigma-Aldrich, St. Louis, MO) supplemented with 10% foetal bovine serum (FCS, Bio&Sell GmbH, Nuremberg, Germany), and 1% antibiotics (Sigma-Aldrich, St. Louis, MO).

### Cell lysate preparation

Cells were harvested and counted to a concentration of 3 × 10^4^ cells/cm^2^ in serum-free DMEM that was aliquoted into reaction tubes. The cell suspensions were then submitted to different treatments to generate necrotic cell lysates. Supernatant control: cell suspension was left at room temperature for 4 h, which is a supernatant and not a lysate that served as control. Sonication lysate: cell suspension was submitted to three cycles of sonication for 15 s and left at room temperature for 3 h; Freeze/thawing lysate: cell suspension was submitted to three cycles of freeze (− 80 °C)/thawing (25 °C) and then left at room temperature for 3 h. After that, the suspensions were centrifuged at 15,000 rcf for 5 min. The supernatants were applied over previously plated cells.

### Experimental setting

Cells were seeded at 3 × 10^4^ cells/cm^2^ into culture plates. The following day, cells were exposed to the undiluted supernatant control, sonication lysate, and freeze/thawing lysate for 20 h before analyses were performed. IL1β and TNFα (ProSpec, Ness-Ziona, Israel) exposure at 20 ng/mL for 20 h was set as a positive control for inflammation induction on cells. All cell lineages were exposed to the respective treatments under standard conditions at 37 °C, 5% CO_2_, and 95% humidity. A toll-like receptor 4 (TLR4) inhibitor, TAK-242 (Merck KGaA, Darmstadt, Germany) at 25 µM, was introduced to verify a possible pathway for inflammation. Also, an interleukin-1 receptor-associated kinase-1/4 inhibitor (Merck KgaA, Darmstadt, Germany) at 15 µM was applied to verify the role of IL1 in the in vitro model generated. Furthermore, to evaluate if the lysates would attach to the surface of materials used in implant dentistry, we treated custom-made grade 4 sand-blasted titanium discs and collagen membranes (BioGide®, Geistlich Pharma AG, Wolhusen, Switzerland) with the cell lysates or the IL1β/TNFα positive setting for 20 h. The materials were washed three times with PBS after exposure to the lysates. Then, cells were seeded over the treated materials and checked for inflammation expression.

### Cell viability assays

For viability experiments, HGF and HSC2 were incubated with the supernatant control, sonication lysate, and freeze/thawing lysate. After 20 h of incubation, MTT (3-[4,5-dimethythiazol-2-yl]-2,5-diphenyltetrazolium bromide; Sigma) solution at a final concentration of 0.5 mg/mL was added to each well of a microtiter plate (CytoOne, Starlab International, Hamburg) for 2 h at 37 °C. The medium was removed and formazan crystals were solubilised with dimethyl sulfoxide. Optical density was measured at 570 nm and the data normalised to unstimulated control values. Cell viability was further confirmed using a Live-dead staining assay kit according to the instructions of the manufacturer (Enzo Life Sciences, Inc., Lausanne, Switzerland).

### Reverse transcription quantitative real-time PCR (RT-qPCR) and immunoassay

For RT-qPCR, after stimulation, total RNA was isolated with the ExtractMe total RNA kit (Blirt S.A., Gdańsk, Poland) followed by reverse transcription (LabQ, Labconsulting, Vienna, Austria) and polymerase chain reaction (LabQ, Labconsulting, Vienna, Austria) on a CFX Connect™ Real-Time PCR Detection System (Bio-Rad Laboratories, Hercules, CA). Primer sequences are shown in Table 1. The mRNA levels were calculated by normalising to the housekeeping gene GAPDH using the ^ΔΔ^Ct method. Supernatants were analysed for IL6 secretion by immunoassay according to the manufacturer’s instruction (R&D Systems, Minneapolis, MN).

**Table 1:** The primer sequences

Primers	Sequence_F (5′ → 3′)	Sequence_R (5′ → 3′)
IL1β IL6	ATGATGGCTTATTACAGTGGCAA GAAAGGAGACATGTAACAAGAGT	GTCGGAGATTCGTAGCTGGA GATTTTCACCAGGCAAGTCT
IL8 GAPDH	AACTTCTCCACAACCCTCTG AGCCACATCGCTCAGACAC	TTGGCAGCCTTCCTGATTTC GCCCAATACGACCAAATCC

### Immunostaining

Immunofluorescent analysis of nuclear factor-kappa B (NF-kB) p65 was performed in HGF and HSC2 cells plated onto Millicell® EZ slides (Merck KgaA, Darmstadt, Germany) at 1 × 10^4^ cells/cm^2^. Cells were submitted to overnight serum starvation and then stimulated with IL1β + TNFα or the cell lysates for 1 h. Cells were fixed with 4% paraformaldehyde (PFA, Sigma-Aldrich, St. Louis, MO), blocked with 1% bovine serum albumin (BSA, Sigma-Aldrich, St. Louis, MO), and permeabilised with 0.3% TritonX-100 (Sigma-Aldrich, St. Louis, MO). Cells were subsequently incubated with NF-kB p65 primary antibody (1:400, Cell Signaling Technology, Cambridge, UK) at 4 °C overnight. Detection was carried out by incubation of Alexa 488 secondary antibody (1:800, Cell Signaling Technology, Cambridge, UK) for 1 h at room temperature. Fluoromount-G mounting media (Invitrogen, Thermo Fisher Scientific, Waltham, MA, USA) containing DAPI was used to mount the glass slides. Images were captured under a fluorescent microscope with a dual excitation filter block DAPI-FITC (Echo Revolve Fluorescence microscope, San Diego, CA) to observe the nuclei translocation.

### Statistical analysis

All experiments were performed at least three times. Statistical analyses of viability, gene expression, and immunoassay were performed with the Friedman test followed by uncorrected Dunn’s test, comparing each experimental group with the unstimulated control. Analyses were performed using Prism v.9 (GraphPad Software, La Jolla, CA). Significance was set at *p* < 0.05 and significant differences between groups were reported with asterisks in the graphs.

## Results

### Cell lysates provoke mild to moderate cell death

**Fig. 1:**
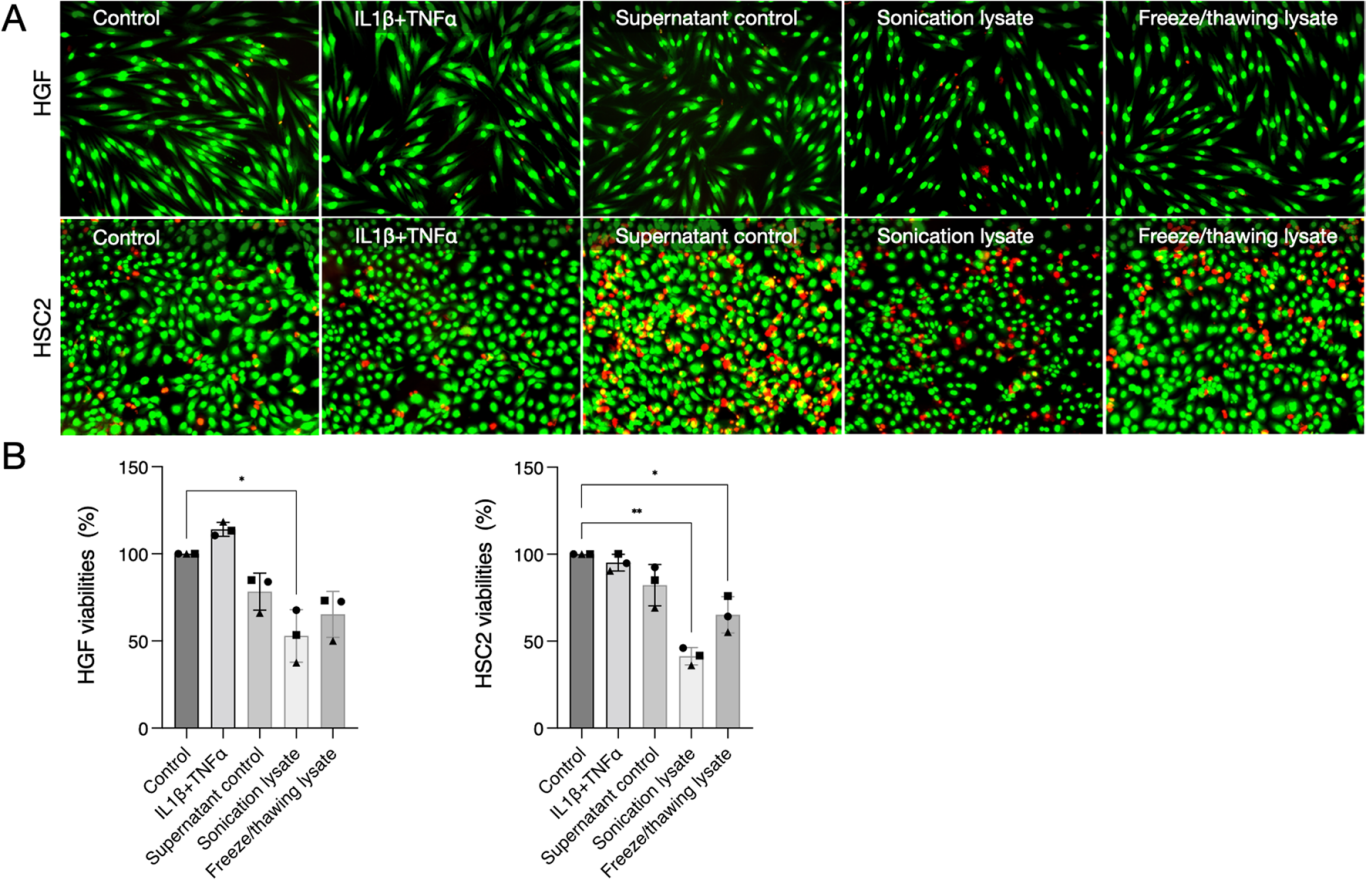
Cell viability was reduced after the exposure of cells to lysates. **A** The Live-dead assay shows the green-stained cells, which are viable, and the red-stained cells, which are dead. **B** The Live-dead results were confirmed by MTT colourimetric test. Sonication lysate provoked a cell viability reduction of about 50% in HGF and HSC2, which was a significant reduction compared to control or non-stimulated cells. The exposure of cells to IL1β and TNFα did not reduce cell viability in gingival fibroblasts and epithelial cells. Different symbol shapes mean independent experiments. * means *p* < 0.05 and ** means *p* < 0.01

To initially evaluate whether the cell lysates could be toxic, we stimulated healthy cells with the different lysates produced. For HGF, we added HSC2 lysates, and HSC2 were challenged with HGF lysates. Then we performed a live-dead assay and MTT. The results show a reduction in the HGF viability when HSC2 lysates were applied, especially regarding lysate produced by sonication, suggesting the damaging potential of epithelial necrotic media over gingival fibroblasts. Similar results of HSC2 viability were found when HGF lysates were applied to these cells (Fig. 1). Sonication lysates provoked a cell viability reduction of about 50% in HGF and HSC2, which was a significant reduction compared to IL1β + TNFα stimulation. Thus, mild to moderate cell damage was observed by introducing cell lysate media over human oral cells.

### HSC2 lysates lead to the expression of IL1β, IL6, and IL8 in gingival fibroblasts

**Fig. 2:**
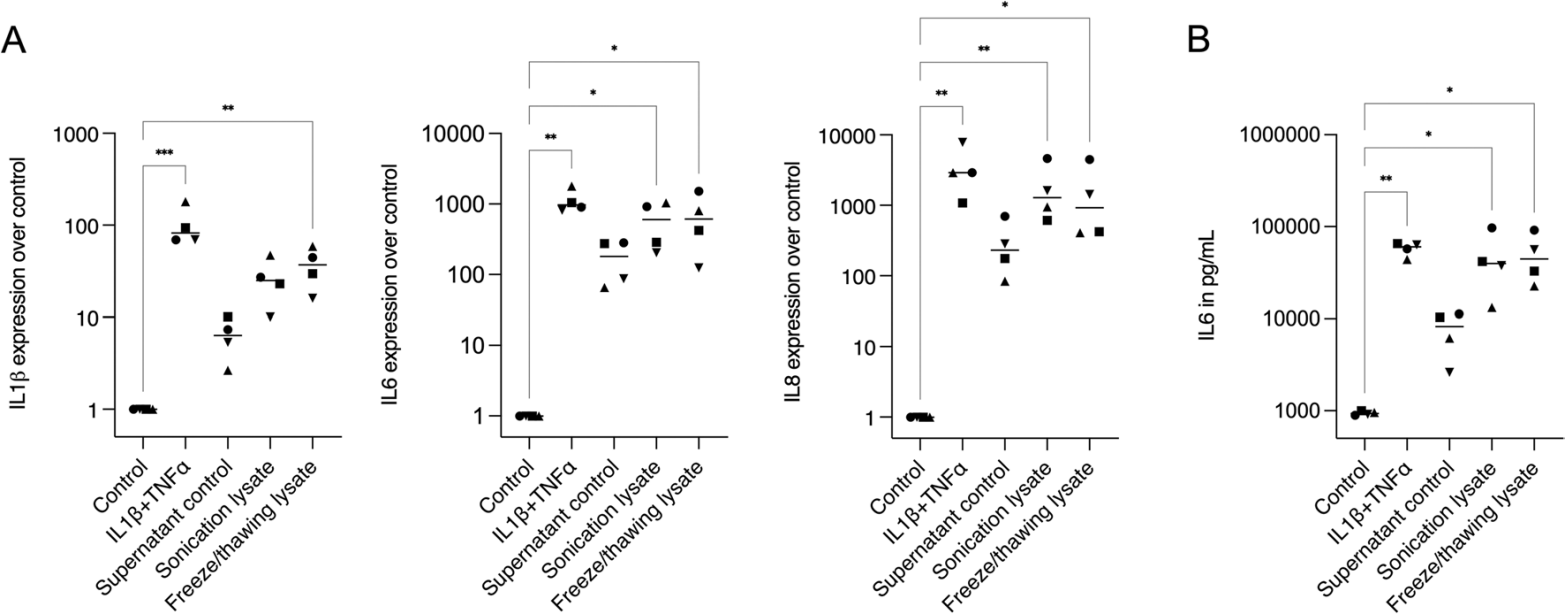
Interleukin expressions of HGF after exposure to IL1β + TNFα treatment or HSC2 lysates. **A** For gene expressions, HGF treated with supernatant control showed significantly less IL1β, IL6, and IL8 expressions than the treatment with the positive control for inflammation (IL1β + TNFα). On the other hand, HGF treated with sonication and freeze/thawing lysates expressed IL1β, IL6, and IL8 to similar levels of the IL1β + TNFα treatment. **B** IL6 immunoassay confirmed the elevated presence of interleukin produced by all groups in this in vitro model for inflammation. In the graphs, different symbol shapes mean independent experiments. * means *p* < 0.05 and ** means *p* < 0.01

To identify potential inflammatory regulation involved after exposure of cells to the lysates, we performed gene expression analyses of IL1β, IL6, and IL8. Accordingly, HSC2 lysates were applied over HGF and the treatment with IL1β and TNFα was set as a positive control for inflammation. We found an impressive expression of IL6 and IL8, and IL1β to a lesser extent (Fig. 2). We compared all the data obtained to the supernatant control or unstimulated cells. Lysates from sonicated and freeze/thawing cells expressed interleukins to a comparable level of IL1β + TNFα treatment, while the supernatant control expressed interleukins at lower levels. The interleukin gene expression was further confirmed by IL6 immunoassay, where again sonication and freeze/thawing lysates produced IL6 at comparable levels to those produced by IL1β + TNFα treatment (Fig. 2). Even though the supernatant control led to less interleukin expression compared to the positive control, the expression and release of interleukins can still be considered high, especially for IL6 and IL8. Together, these results demonstrate that HSC2 necrotic lysates provoke an intense inflammatory response in gingival fibroblasts.

**Fig. 3:**
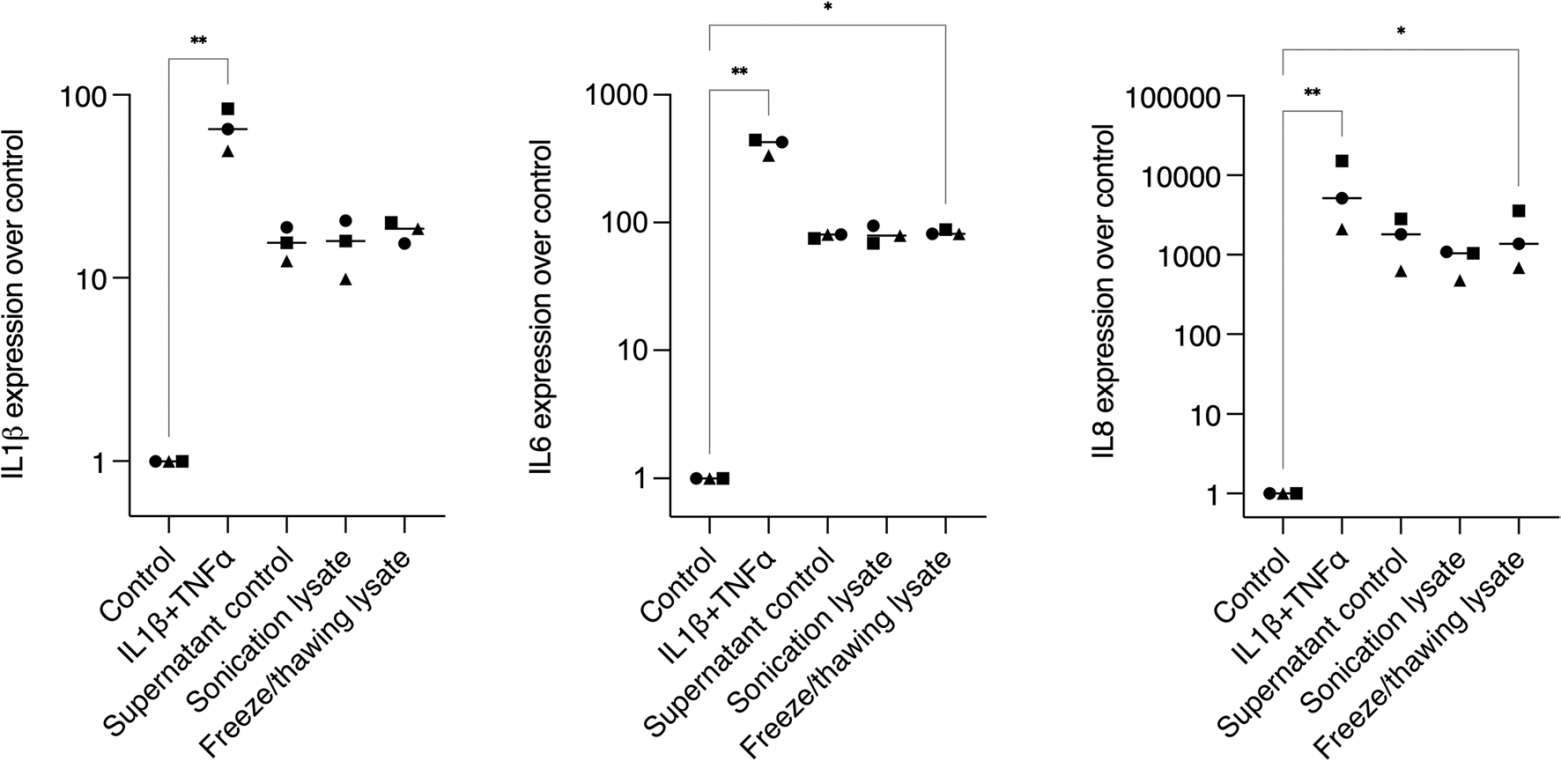
Interleukin expressions of HGF after exposure to IL1β + TNFα treatment or TR146 lysates. Supernatant control, sonication lysate, and freeze/thawing lysate led to a potent expression of IL8, while the expression of IL6 and especially IL1β was lower. In the graphs, different symbol shapes mean independent experiments. * means *p* < 0.05 and ** means *p* < 0.01

To further confirm the inflammatory potential of epithelial cells over gingival fibroblasts, we introduced a different epithelial cell line. Hence, TR146 lysates were applied over HGF overnight, again using IL1β and TNFα as a positive control for inflammation. A strong expression of IL8 was observed for all cell treatments, followed by IL6 and IL1β to a lesser extent (Fig. 3). In this setting, however, no differences were observed in the level of interleukins expression among the different cell lysates for IL1β, but freeze/thawing lysate provoked stronger IL6 and IL8 expressions when compared to unstimulated cells.

Still, to ratify that the inflammatory response was from the necrotic cells and not from possible contamination or impairment of the conditioning media, we prepared aliquots of serum-free media and submitted them to the same procedures performed for the cell lysates preparation, i.e. no treatment, sonication, and freeze/thawing cycles followed by 3 h of incubation at room temperature. None of those prepared media led to the expression of interleukins by HGF (data not shown), suggesting that the applied method did not impair the standard culturing media. High temperature (75 °C and 100 °C) treatments were also tested resulting in weak interleukin expression outcomes, suggesting that the molecules of interest are heat sensitive (data not shown).

### Necrotic gingival fibroblasts did not increase the expression of IL1β, IL6, and IL8 in epithelial cells

**Fig. 4:**
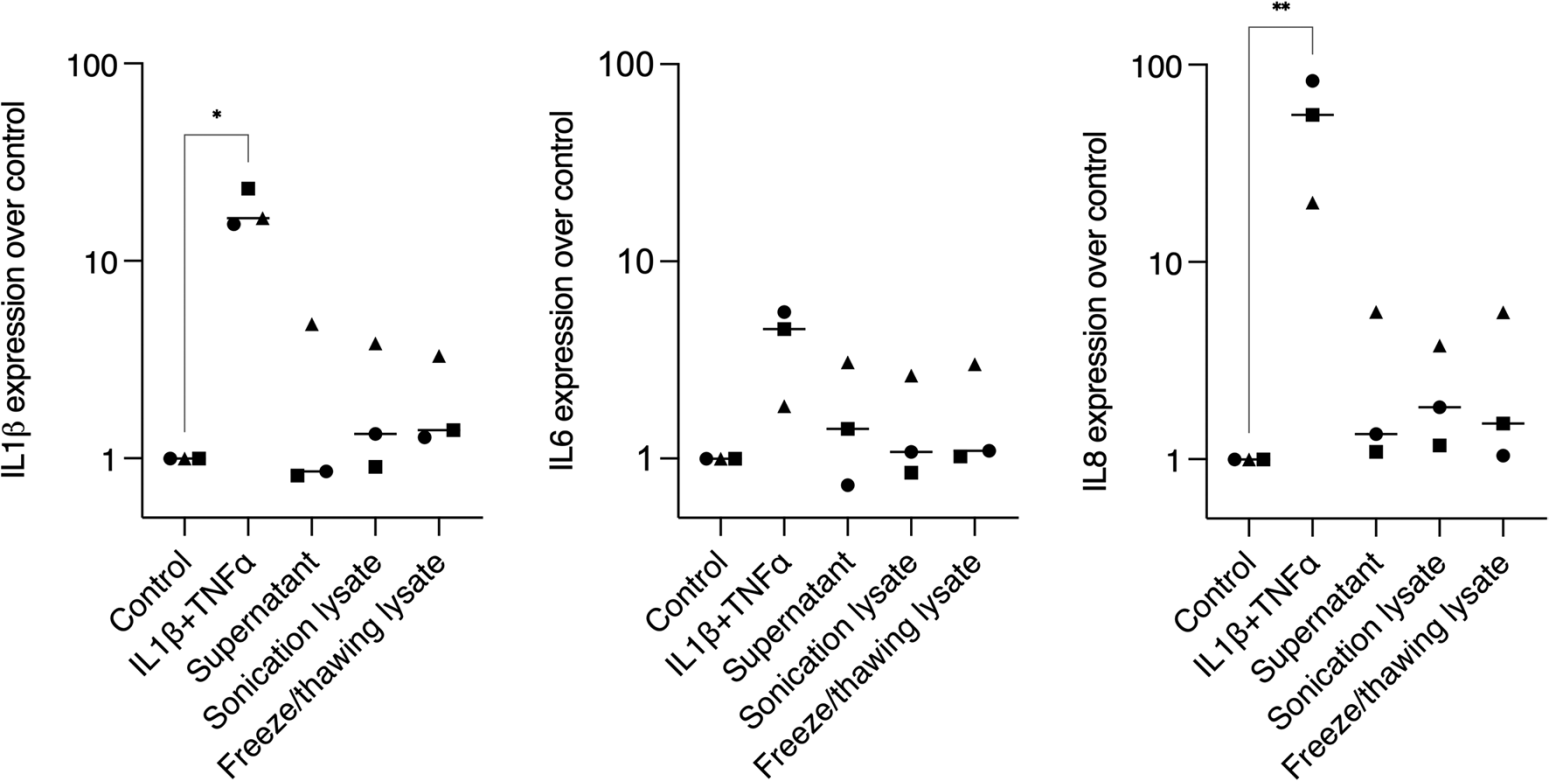
Interleukin expressions of HSC2 after exposure to IL1β + TNFα or HGF lysates. Supernatant control, sonication lysate, and freeze/thawing lysate led to a weak expression of IL1β, IL6, and IL8, while the positive control for inflammation (IL1β + TNFα treatment) led to modest interleukin expressions, with remarks to IL8. In the graphs, different symbol shapes mean independent experiments. * means *p* < 0.05 and ** means *p* < 0.01

To further explore whether the opposite setting would lead to an inflammatory response, we tested HGF lysates on HSC2 cells for overnight stimulation, using IL1β and TNFα as positive control for inflammation. The data show that IL1β + TNFα treatment led to a convincing expression of IL8, and IL1β and IL6 to a lesser extent. In contrast to the HGF findings, however, the HGF lysates on HSC2 cells did not produce a robust expression of interleukins (Fig. 4). IL6 immunoassay confirmed the lack of IL6 release from HSC2 cells (data not shown). Additionally, no differences were observed in the level of interleukin expression among the different cell lysates.

### Epithelial necrotic cells lead to nuclear translocation of p65 in gingival fibroblast, suggesting inflammation

**Fig. 5:**
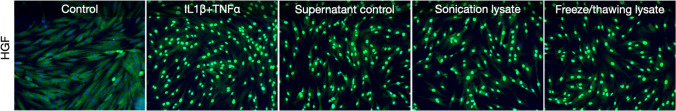
Immunofluorescence of NF-kB p65 in human gingival fibroblasts. The images show that IL1β + TNFα, supernatant control, sonication lysate, and freeze/thawing lysate stimuli led to nuclear translocation of p65, suggesting inflammation of cells through the activation of the NF-κB signalling pathway, which does not occur for unstimulated cells (control)

The combined IL1, IL6, and IL8 expression may suggest that necrotic epithelial cells induce the exacerbated release of inflammatory cytokines via Toll-like receptors (TLR) and NF-kB in gingival fibroblasts. As part of the NF-κB signalling pathway, p65 is typically involved in the inflammatory response. This pathway can be induced by stressful stimuli, such as IL1β, TNFα, and necrotic cells. Therefore, to additionally examine the epithelial lysates’ potential for HGF inflammation, we performed immunostaining of NF-kB p65 to observe the nuclear translocation of cells following stimulation. In unstimulated cells (control), NF-κB subunits are restricted to the cytoplasm due to the inhibitory effects of the inhibitor of kB (IkB), which selectively binds to the p65 heterodimer and masks their nuclear localisation signal, preventing their nuclear translocation. Likewise, Fig. 5 shows that unstimulated cells had the DAPI (blue staining) differentiating the nuclei from the cytoplasm in green (no nuclear translocation). In contrast, IL1β + TNFα, supernatant control, sonication lysate, and freeze/thawing lysate stimuli induced NF-κB signalling through activating the IκB kinase (IKK) complex, which pushes p65 to locate in the nucleus (nuclear translocation), and the nuclei then appear overlapped in green. This activation of NF-κB signalling suggests inflammation of cells, which agrees with the gene expression and immunoassay results.

Furthermore, a TLR4 inhibitor (TAK-242) and an IL1 inhibitor were introduced to this in vitro setting. However, those inhibitors did not successfully block interleukin expression (Supplement Fig. 1), suggesting that pathways of inflammation other than TLR4 and IL1 are involved in the model we established herein.

### Epithelial lysates can attach to titanium and collagen surfaces and provoke IL8 expression in gingival fibroblasts

**Fig. 6:**
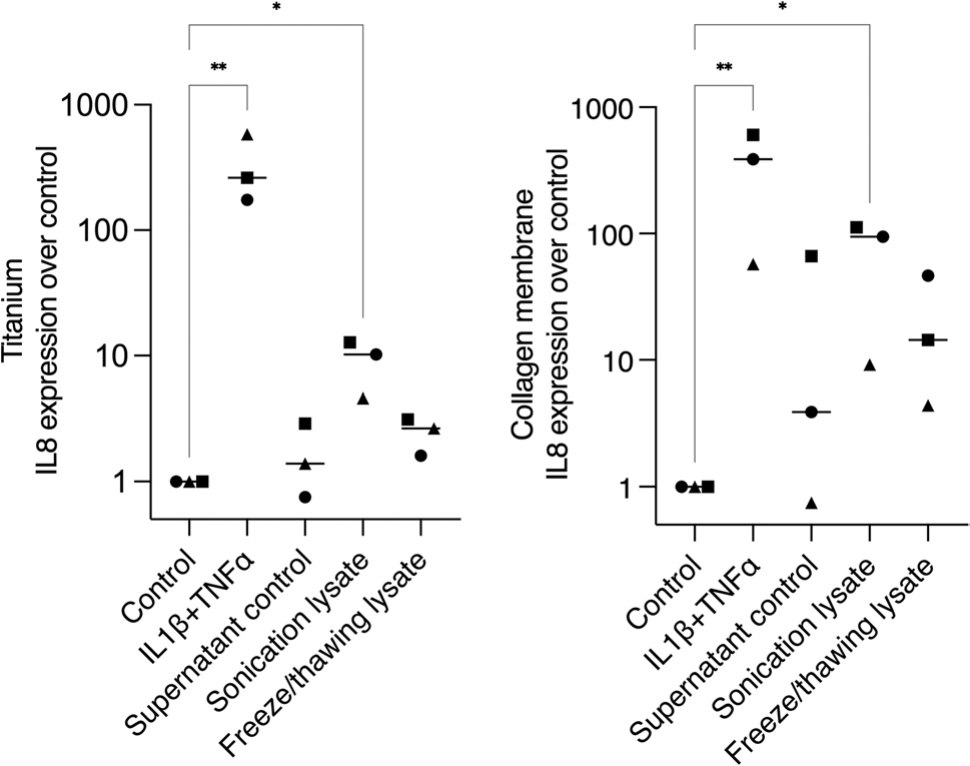
Interleukin-8 expressions of HGF seeded over titanium and collagen surfaces exposed to IL1β + TNFα or HSC2 lysates. The highest IL8 expression was seen for IL1β + TNFα for titanium and collagen materials, followed by sonication lysate stimulation. In the graphs, different symbol shapes mean independent experiments. * means *p* < 0.05 and ** means *p* < 0.01

Finally, we speculated if the lysates would attach to material surfaces, such as rough titanium and collagen membranes, which are routinely used in implant dentistry and thus exposed to cells that were damaged during surgical procedures. Hence, we exposed the materials to the HSC2 lysates for 20 h. After incubation, the materials were washed three times with PBS, and HGF were seeded over the material samples. The next day, samples were transferred to a new plate to eliminate the confounding factor of cells attached to the plate and not the material, and then RNA extraction proceeded. IL8 gene expression showed that the HSC2 lysates could adhere to the materials and provoke the inflammatory reaction of HGF (Fig. 6).

## Discussion

Clinically, the integrity of the oral epithelial barrier is of utmost importance as it offers protection to the underlying connective tissue, which is more sensitive to environmental damage and bacterial invasion [2, 3, 4, 6]. The loss of integrity of the epithelial cell layer caused by a catabolic inflammatory environment, bacterial proteases, or mechanical damage is associated with inflammatory responses of the underlying connective tissue, which is mainly consisted of gingival fibroblasts but also immune cells, including macrophages [5, 6, 7]. However, whether and how the damage of oral epithelial cells signals the demand for a local repair by initiating an inflammatory response of the adjacent connective tissue remains largely unknown. In this study, we report that the lysates obtained upon sonication or freeze/thawing of oral squamous cell carcinoma cells provoked a robust increase in the expression of interleukins IL1, IL6, and IL8 by gingival fibroblasts, which was confirmed by immunoassays of IL6. Conversely, lysates obtained from the gingival fibroblasts failed to increase the expression of interleukins in oral squamous cell carcinoma cells. Additionally, the epithelial cell lysates caused the activation of the NF-kB signalling cascade in gingival fibroblasts as indicated by the nuclear translocation of p65, suggesting inflammation. Finally, we demonstrated that the epithelial cell lysates can adhere to the titanium and particularly to collagen membrane surfaces indicated by the IL8 expression of gingival fibroblasts.

Clinically, dying cells may represent a threat to the body, for instance, in an evolving cancer that may be eliminated by the host immune response [21]. On the other hand, the inflammatory response may counteract certain pathological processes; for example, in an ischemic infarct, where inflammation-induced vasodilatation may help perfuse adjacent ischemic areas [22, 23]. In other forms of injury, increased blood flow and fluid leakage may help dilute and drain away soluble injurious agents or the cellular response may encase offending particulate material, e.g. in a granuloma [22]. Inflammation also initiates tissue repair and regeneration, then promoting the healing of damaged or lost tissues [24]. Thus, even in these non-bacterial situations, the inflammatory response may remain defensive and limit further damage to the host [22]. However, the inflammatory response, although beneficial in most cases, may impair the host tissues, especially when chronic. This may happen in cases of continuing mechanical damage to tissues.

The reduced inflammatory response of epithelial cells challenged by gingival fibroblast lysates is evidence of the resistance of epithelial cells against external dangers and further confirmation of their protective function. Similar to our finding, cell-penetrating peptides (CPP) failed to activate NF-κB and no significant increase in the release of the IL6 and IL8 was detected in epithelial cells exposed to these CPP complexes [25]. Nevertheless, sonication lysate provoked moderate toxicity by reaching about 50% reduced cell viability. This means that the necrotic gingival cells were able to reduce the epithelial cell viability but failed to provoke an inflammatory response. This could be related to the potential of these lysates to affect the epithelial barrier by modifying the expression and integrity of the different cell–cell junctions [6]. To sustain their function, the stratified epithelia of the oral mucosa have to sustain tight cell–cell adhesion in the viable cells that involve intercellular tight and adherent junctions which connect to the actin cytoskeleton [6]. While we focused on the inflammatory response, further studies could then report on the expression of these adhesion molecules under the challenge of necrotic cell lysates.

Primary inflammatory stimuli, including microbial products and cytokines such as IL1β, IL6, and TNFα, mediate inflammation through interaction with receptors, e.g. the TLRs and IL1 receptor (IL1R) [26, 27]. Receptor activation triggers important intracellular signalling pathways, including the nuclear factor kappa-B (NF-κB) signalling [26]. NF-kB controls the expression of the molecules needed for acute inflammation, especially the pro-inflammatory cytokines tested herein, i.e. IL1, IL6, and IL8 [22]. However, blocking of interleukin-1 receptor-associated-kinase-1/4 inhibitor and the TLR4 inhibitor TAK-242 could not reduce the inflammatory activity of the HSC2 cell lysates. Together with our observations that HGF and HSC2 lysates failed to cause inflammation in RAW 264.7 macrophages (data not shown), these data suggest that inflammation pathways are not caused by HSC2-derived IL1 or any LPS-related contamination of the lysates. Nevertheless, future studies could consider other TLRs, such as TLR2 or TLR9, that can activate NF-kB signalling [22]. The use of TLR2 or TLR9 antagonists such as MMG-11 [28] and inhibitory oligonucleotides (ODN) [29], respectively, could prove this potential involvement. Thus, we could not explain what pro-inflammatory molecules the epithelial cells release and what receptor-mediated signalling pathway fibroblasts use to drive the inflammatory response.

Antioxidant defence systems, including antioxidant enzymes, influence the oxidative stress [26]. Elevated oxidative stress can produce reactive oxygen species (ROS) [26]. Herein, we checked the ROS release for the gingival fibroblasts stimulated with the HSC2 lysates; however, the stimulation did not lead to ROS release (data not shown). Likewise, we hypothesised if the HSC2 cell lysates could activate the NLRP3 inflammasome, which is related to a potent inflammatory response due to the maturation and release of IL1β and IL18 [16, 20, 30]. The NLRP3 activation is closely related to the activation of Caspases, especially Caspases-1 and -11, which in turn will cleave the membrane pore-forming molecule known as Gasdermin D, in a cascade process called pyroptosis [30, 31]. It is the Gasdermin D that produces pores into the cell membrane that will allow the release of the IL1β and IL18 that were maturated due to the action of the above-mentioned caspases [30, 31]. Nonetheless, the HSC2 lysates failed to increase the expression of NLRP3 and the related genes, namely Caspase-1, Caspase-11, Gasdermin D, and IL18 in gingival fibroblasts (data not shown). Therefore, the hypothesis for pyroptosis signalling involvement can be rebutted.

The use of titanium and collagen membranes was proposed to add to the clinical relevance of this in vitro study since these materials are used in implant dentistry surgical procedures, when cells are largely damaged. We showed that necrotic epithelial cell lysates produced by sonication could attach to the surfaces and provoke the gene expression of IL8 by gingival fibroblasts. This can be extended to dental surfaces, where we know that virulence factors can adhere to and provoke inflammation [32]. Therefore, the overall recommendation is to avoid as much as possible the injuries of the oral epithelium as cell fragments can adhere to surfaces and cause an inflammatory reaction. Minimal invasive approaches such as flapless surgeries and guided surgeries help to reduce tissue damage and prevent further inflammatory triggering. It should be noted, however, that the preliminary data we show are more of a primer pointing towards the demand for future more translational research aiming to understand how necrotic cells affect the behaviour of biomaterials in dentistry but also in other fields of regenerative medicine.

This study has all the limitations of an in vitro study. The main restraint is the translation of the outcomes to the clinical relevance. Nevertheless, our hypothesis that injured epithelial cells or fragments impair gingival fibroblasts seems to be tangible considering the continuous mechanical challenges affecting the oral mucosa. Impressively, as far as we know, no one tested this setting before, which opens a path for further in vivo research. However, the better establishment of the in vitro outcomes should be consolidated before moving to translational research. In this sense, as previously mentioned, future in vitro studies could thus consider other TLR antagonists to confirm the likelihood of the NF-kB signalling involvement of the present model. Additionally, MAPK and JAK-STAT pathways for inflammation could also be assessed. Moreover, in-depth analyses to verify what are the active contents released by the disrupted necrotic cells would give insights into the downstream inflammatory response generated by challenging healthy cells with necrotic cells. In addition, how the lysates attach to surfaces should be addressed in further research. Finally, cell lysates without centrifugation, since the lysates presumably contain fragments of the cell membranes, could be tested to analyse the effects of the necrotic cell fragments on healthy cells. Another drawback of this study is that we have not used primary human oral epithelial cells but epithelial cells originating from squamous carcinoma. Thus, our conclusions are restricted to those cell lines and not oral epithelial cells in general. Furthermore, the application of other cell lines, particularly inflammatory cell lineages such as macrophages and monocytes, must be considered. Although this study has limitations, the suggestions for future studies are exciting as the results may lead to contributions to the knowledge of the pathobiology of mechanical damage, i.e. aseptic inflammation caused by mastication or iatrogenic damage.

In summary, at sites of tissue injury, damaged epithelial cells release factors that trigger the inflammatory cascade, along with chemokines and growth factors, which attract neutrophils and monocytes [26]. The release of interleukins and chemokines by gingival fibroblasts is evidence that they become inflammatory cells in response to external dangers, in the present case, in response to epithelial necrotic cells. Therefore, the overall main conclusion we may draw from this in vitro research is that injured oral epithelial cells can release factors that incite gingival fibroblasts to become pro-inflammatory cells.

## Supplementary Information

ESM 1 (PNG 637 kb)

High Resolution Image (TIFF 704 kb)

## Data Availability

Not applicable.
