# Supplementary figures and images for: Oral squamous carcinoma cell lysates provoke exacerbated inflammatory response in gingival fibroblasts
Source: Clin Oral Investig. 2023 Jun 30;27(8):4785–94. doi: 10.1007/s00784-023-05107-x (PMC10415472; doi:10.1007/s00784-023-05107-x)

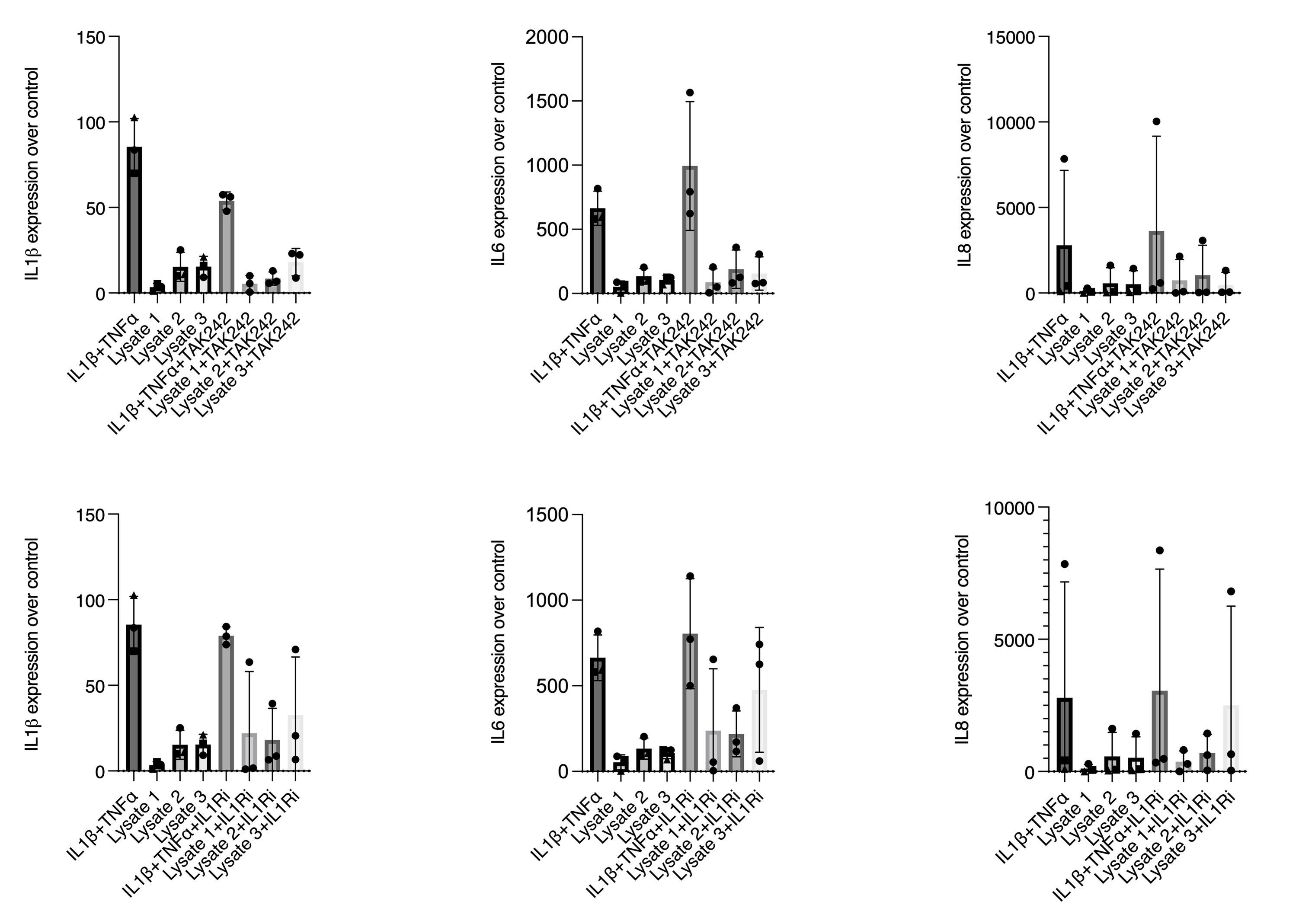

Supplement: Supplementary file 1 — (PNG 637 kb) [file 784_2023_5107_Fig7_ESM.png]

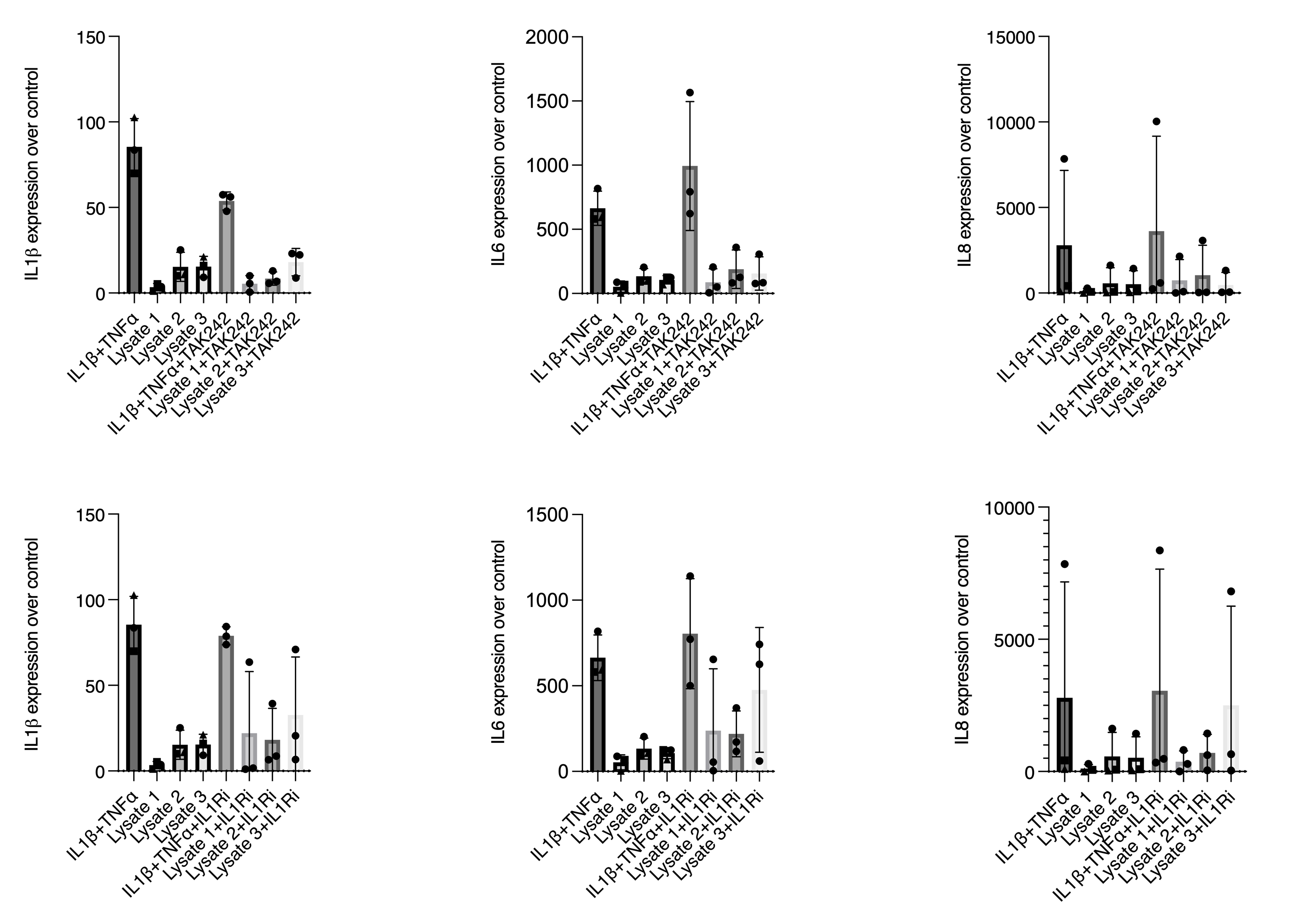

Supplement: Supplementary file 2 — High Resolution Image (TIFF 704 kb) [file 784_2023_5107_MOESM1_ESM.tiff]
